# AT excursion: a new approach to predict replication origins in viral genomes by locating AT-rich regions

**DOI:** 10.1186/1471-2105-8-163

**Published:** 2007-05-21

**Authors:** David SH Chew, Ming-Ying Leung, Kwok Pui Choi

**Affiliations:** 1 Department of Statistics and Applied Probability, National University of Singapore, Singapore 117546, Singapore; 2 Department of Mathematical Sciences and Bioinformatics Program, The University of Texas at El Paso, TX 79968, USA; 3 Department of Mathematics, National University of Singapore, Singapore 117543, Singapore

## Abstract

**Background:**

Replication origins are considered important sites for understanding the molecular mechanisms involved in DNA replication. Many computational methods have been developed for predicting their locations in archaeal, bacterial and eukaryotic genomes. However, a prediction method designed for a particular kind of genomes might not work well for another. In this paper, we propose the AT excursion method, which is a score-based approach, to quantify local AT abundance in genomic sequences and use the identified high scoring segments for predicting replication origins. This method has the advantages of requiring no preset window size and having rigorous criteria to evaluate statistical significance of high scoring segments.

**Results:**

We have evaluated the AT excursion method by checking its predictions against known replication origins in herpesviruses and comparing its performance with an existing base weighted score method (BWS_1_). Out of 43 known origins, 39 are predicted by either one or the other method and 26 origins are predicted by both. The excursion method identifies six origins not predicted by BWS_1_, showing that the AT excursion method is a valuable complement to BWS_1_. We have also applied the AT excursion method to two other families of double stranded DNA viruses, the poxviruses and iridoviruses, of which very few replication origins are documented in the public domain. The prediction results are made available as supplementary materials at [1]. Preliminary investigation shows that the proposed method works well on some larger genomes too.

**Conclusion:**

The AT excursion method will be a useful computational tool for identifying replication origins in a variety of genomic sequences.

## Background

Recent advances in biotechnology have rendered sequencing a complete genome routine. With the increasing availability of DNA sequences, computational methods to predict likely locations of important functional sites before experimental search are highly valuable because the computational predictions can often help design finely tuned experiments to find these functional sites in shorter time with less labor and fewer resources. Replication origins, which are places on the DNA molecules where replication processes are initiated, are considered important sites for understanding the molecular mechanisms involved in DNA replication. For some viruses with double stranded DNA (dsDNA) genomes in particular, detailed knowledge of their replication processes have had significant impact in developing effective strategies to control the growth and spread of viruses (see, for example, [2]).

A number of computational methods have been developed for predicting replication origins in bacterial, archaeal, and eukaryotic genomes. All these algorithms exploit certain characteristic sequence features found around the replication origins. For example, Lobry [3] employs the GC skew plot to predict replication origins and terminus in bacterial genomes. The skew (G-C)/(G+C), where G and C respectively stand for the percentages of guanine and cytosine bases in a sliding window, switches polarity in the vicinity of the replication origin and terminus, with the leading strand manifesting a positive skew. Salzberg et al. [4] predict the replication origins for a number of bacterial and archaeal genomes by identifying some 7-mers and/or 8-mers whose orientation is preferentially skewed around the replication origins. Zhang and Zhang [5] use the Z-curve method successfully to identify several replication origins in bacterial and archaeal genomes. The Z-curve of any given DNA sequence is a three-dimensional curve which uniquely represents the sequence so that unusual sequence compositional features, such as those around a replication origin, can sometimes be visually recognized. Mackiewicz et al. [6] propose three methods, based on DNA asymmetry, the distribution of DnaA boxes and dnaA gene location, were applied to identify the putative replication origins in 112 bacterial chromosomes. They find that DNA asymmetry is the most universal method of putative oriC identification and better prediction can be achieved when the method is applied together with others.

For eukaryotic DNA, Breier et al. [7] develop the Oriscan algorithm to predict replication origins in the S. cerevisiae genome by searching for sequences similar to a training set of 26 known yeast origins pinpointed by site-directed mutagenesis. Oriscan uses both the origin recognition complex binding site and its flanking regions to identify candidates, and then ranks potential origins by their likelihood of activity. More recently, wavelet based multi-scale analysis of DNA strand asymmetries have also been developed [8,9] for detecting mammalian DNA replication origins.

It is important to note that a prediction method designed for one kind of genomes may not necessarily work well on others because the differences in DNA replication mechanisms in different organisms naturally lead to differences in sequence features around their replication origins. One would not expect that the prediction methods designed for bacterial, archaeal, and eukaryotic genomes can be applied directly to viral genomes and produce accurate results. Indeed, when we attempted to use the above algorithms on some herpesviruses genomes with known replication origins like those listed in Table 5 of [10], a variety of difficulties were encountered. For instance, no clear cut switches of polarity were observed in the GC skew plot. No definitive peaks can be visually identified from the Z-curves as potential replication origins of the viruses. When we mined for DnaA boxes [6] in the herpesviruses, just one cluster of DnaA boxes was observed, but it is not near to any known replication origins. Information about origin recognition complex binding sites for herpesvirus genomes, needed for applying Oriscan, are not readily available. While the method based on oligomers skew [4] is designed to work for genomes with single replication origins, the herpesviruses and many other dsDNA viruses contain multiple replication origins in their genomes. 

Computational prediction of replication origins, based on the observation of a high concentration of palindromes around the origins, for dsDNA viral genomes was first attempted by Masse et al. [11] on the human cytomegalovirus. Leung et al. [10] formalize the procedure by laying down the mathematical foundation to justify the use of scan statistics for identifying statistically significant palindrome clusters. The location of such palindrome clusters are then taken to be the likely locations of replication origins in herpesviruses. Viewing the scan statistics approach as equivalent to counting the palindromes in sliding windows, Chew et al. [12] offer two more refined schemes of quantifying palindrome concentration to improve the sensitivity of the prediction. One of these schemes, namely the base weighted scheme (BWS_1_), which scores each palindrome according to how rarely it is expected to occur in a nucleotide sequence generated randomly as a first order Markov chain, is found to be the most sensitive for the herpesviruses.

Because of the lack of strong family-wide sequence similarities around the origins, the above prediction methods designed for relatively large and complex dsDNA viruses like the herpesviruses with over 100,000 base pairs in the genomes are based on various sequence statistics rather than the actual nucleotide sequences around replication origins.

Herpesviruses utilize two different types of replication origins during lytic and latent infections. For each type of origins, the count and locations in the genome vary from one kind of herpesvirus to another. Most herpesviruses have one to two copies of latent and lytic origins. It has been documented in various studies (e.g. [11,13,14]) that the nucleotide sequences around the replication origins are specific to the individual viruses. Yet the presence of clusters of direct or inverted repetitive sequences, including palindromes, is quite common in both types of origins in many members of the herpesvirus family (see [12] and references therein).

Lin et al [15] have observed that in some herpesvirus genomes, the nucleotide sequences around replication origins are richer in A and T bases. This is not surprising because DNA replication typically requires the binding of an assembly of enzymes (e.g., helicases) to locally unwind the DNA helical structure, and pull apart the two complementary strands (see Chapter 1 in [16,17]). Higher AT content around the origins makes the two complementary DNA strands bond less strongly to each other. This facilitates the two strands to be pulled apart and initiate the replication process. Indeed, Segurado et al. [18] have used a sliding window approach to find "islands" within the *Schizosaccharomyces pombe *genome that have high AT content. They measure base composition using sliding windows of different sizes and find that AT content of windows in regions containing replication origins are significantly higher than those that do not. 

Chew et al. [12] have also reported using sliding windows of AT percentages on herpesviruses. Using windows with top AT percentages they are able to predict 65% of replication origins in their dataset.  Moreover, this method has successfully identified four origins not predicted by BWS_1_, suggesting that the AT percentages may be a useful sequence feature to be incorporated into the set of replication origin prediction tools for dsDNA viruses. This motivates us to seek a means to better quantify the AT content variation in genome sequences. We find that the general score based excursion approach first proposed by Karlin and Altschul in [19] fits our purpose very well when it is applied appropriately to quantify local AT abundance. The excursion approach has the advantages of not requiring a preset sliding window size and having rigorous criteria to evaluate statistical significance of high scoring segments [20-22].

There are three main objectives in this paper. First, we shall develop the AT excursion method as a possible alternative to existing approaches for replication origin prediction in DNA sequences. Second, we shall assess the performance of AT excursion in comparison with the prediction results of BWS_1 _on a data set of currently known origins of the herpesviruses. The herpes family is chosen as it is one of the bigger families of viruses with known replication origins so that the performance of our prediction method can be assessed. Our results demonstrate that the AT excursion method not only can compare with but can also complement the BWS_1 _predictions very well. Having established that AT excursion method is a credible prediction tool, our third objective is to use it for predicting likely replication origin locations for two other families of dsDNA viruses, namely the poxviruses and iridoviruses of which very few replication origins are documented in the public domain. To demonstrate the generality of the AT excursion approach, we also apply it to several larger genomes.

## Methods

We adopt the score-based excursion approach [19] to identify segments of a genome having high AT concentration. This, in turn, forms the basis of our proposed method to predict replication origins for the herpesviruses. Table 1 presents the viruses to be analyzed. The data set comprises all complete genome sequences of the herpesvirus family downloaded from GenBank at the NCBI web site in March 2006. For each virus, we list its abbreviation, accession number, sequence length, and AT percentages.

**Table 1:** The list of herpesviruses to be analyzed.

**Virus**	**Abbrev.**	**Accession**	**Length**	**AT%**
Alcelaphine herpesvirus 1	alhv1	NC_002531	130608	53
Ateline herpesvirus 3	athv3	NC_001987	108409	63
Bovine herpesvirus 1	bohv1	NC_001847	135301	28
Bovine herpesvirus 4	bohv4	NC_002665	108873	59
Bovine herpesvirus 5	bohv5	NC_005261	138390	25
Callitrichine herpesvirus 3	calhv3	NC_004367	149696	51
Cercopithecine herpesvirus 1	cehv1	NC_004812	156789	26
Cercopithecine herpesvirus 2	cehv2	NC_006560	150715	24
Cercopithecine herpesvirus 8	cehv8	NC_006150	221454	51
Cercopithecine herpesvirus 9	cehv7	NC_002686	124138	59
Cercopithecine herpesvirus 15	cehv15	NC_006146	171096	38
Cercopithecine herpesvirus 16	cehv16	NC_007653	156487	24
Cercopithecine herpesvirus 17	mmrv	NC_003401	133719	47
Equid herpesvirus 1	ehv1	NC_001491	150224	44
Equid herpesvirus 2	ehv2	NC_001650	184427	43
Equid herpesvirus 4	ehv4	NC_001844	145597	50
Gallid herpesvirus 1	gahv1	NC_006623	148687	52
Gallid herpesvirus 2	gahv2	NC_002229	174077	56
Gallid herpesvirus 3	gahv3	NC_002577	164270	46
Human herpesvirus 1	hsv1	NC_001806	152261	32
Human herpesvirus 2	hsv2	NC_001798	154746	30
Human herpesvirus 3	vzv	NC_001348	124884	54
Human herpesvirus 4	ebv	NC_007605	171823	41
Human herpesvirus 5 (AD169)	hcmv	NC_001347	230287	43
Human herpesvirus 5 (Merlin)	hcmv-m	NC_006273	235645	42
Human herpesvirus 6	hhv6	NC_001664	159321	58
Human herpesvirus 6B	hhv6b	NC_000898	162114	58
Human herpesvirus 7	hhv7	NC_001716	153080	63
Human herpesvirus 8	hhv8	NC_003409	137508	47
Ictalurid herpesvirus 1	ichv1	NC_001493	134226	43
Meleagrid herpesvirus 1	mehv1	NC_002641	159160	52
Murid herpesvirus 1	mcmv	NC_004065	230278	41
Murid herpesvirus 2	rcmv	NC_002512	230138	39
Murid herpesvirus 4	muhv4	NC_001826	119450	53
Macaca fuscata rhadinovirus	mfrv	NC_007016	131217	48
Ostreid herpesvirus 1	oshv1	NC_005881	207439	61
Ovine herpesvirus 2	ohv2	NC_007646	135135	47
Pongine herpesvirus 4	ccmv	NC_003521	241087	38
Psittacid herpesvirus 1	pshv1	NC_005264	163025	39
Saimiriine herpesvirus 2	sahv2	NC_001350	112930	65
Suid herpesvirus 1	shv1	NC_006151	143461	26
Tupaiid herpesvirus 1	thv	NC_002794	195859	34

### Score-based sequence analysis

Score-based sequence analysis is a powerful and yet flexible tool to identify segments of a biological (DNA, RNA or amino acids) sequence containing high concentration of residues of interest according to the users' objectives. One assigns high positive scores to residues of interest, high negative scores to contrasting residues and low or zero scores for the rest. Using various score schemes, Karlin and his collaborators applied this approach with success to gene finding, identification of transmembrane protein segments, and DNA-binding domains. For details and other applications, see, for example, [20-22] and the references therein.

Our interest in this paper is to identify segments of genomic sequences with high AT content. Towards this end, we label bases C or G as "strongly bonding" base S; and bases A or T as "weakly bonding" base W. Under this label, S bases (i.e., C or G) are given a score of *s *and W bases (i.e., A or T) a score of *w*. The scores *s *and *w *will be specified below. We next model the genomic sequence as a realization of a sequence of independent and identically distributed random variables, *X*_1_, *X*_2_, ..., *X*_*n *_(where *n *is the genome length), taking values in {*s*, *w*}. If the *i*th base is labeled as W, *X*_*i *_is given a score *w *otherwise *X*_*i *_= *s*. We let *p *:= *P*(*X*_*i *_= *s*) and *P *(*X*_*i *_= *w*) = 1 - *p *(denoted by *q*). The parameter *p *is naturally estimated by the CG percentage in the genome. An additional constraint needed to be imposed on the choice of *s *and *w *is that the expected score per base *μ *= *ps *+ *qw *has to be negative. This condition prevents favoring long segments to be high scoring segments. A moment's reflection shows that we can always standardize one of the scores to be 1. Here we let *w *= 1 and choose *s *to be a negative integer (integer-value choice due to a technical reason as pointed out after equation (3)) so that the expected score per base, *μ *= *ps *+ *qw *is close to the value of -0.5 (where we adopt Karlin's choice of expected value as in [21]). In other words, *w *:= 1 and

s:=⌊μ−qwp⌋,
 MathType@MTEF@5@5@+=feaafiart1ev1aaatCvAUfKttLearuWrP9MDH5MBPbIqV92AaeXatLxBI9gBaebbnrfifHhDYfgasaacH8akY=wiFfYdH8Gipec8Eeeu0xXdbba9frFj0=OqFfea0dXdd9vqai=hGuQ8kuc9pgc9s8qqaq=dirpe0xb9q8qiLsFr0=vr0=vr0dc8meaabaqaciaacaGaaeqabaqabeGadaaakeaacqWGZbWCcqGG6aGocqGH9aqpdaGbdaqaamaalaaabaacciGae8hVd0MaeyOeI0IaemyCaeNaem4DaChabaGaemiCaahaaaGaayj84laawUp+aiabcYcaSaaa@3D1A@

where *μ *= -0.5 and ⌊·⌋ denotes the integer floor function.

### Excursions and their values

We next compute the cumulative scores and seek to identify segments of the genome that have significantly high scores. As we are only interested in segments with positive additive scores, we reset our cumulative scores to zero whenever it becomes negative.

The *excursion scores E*_*i*_'s are defined recursively as

*E*_0 _= 0, *E*_*i *_= max{*E*_*i*-1 _+ *X*_*i*_, 0}, for 1 ≤ *i *≤ *n*.

Using this recursive definition, we are able to construct "excursions" for each of the genomes. An *excursion *starts at a point *i *where *E*_*i *_is zero and ends at *j > i *where *E*_*j  *_is the very next zero. The score then stays at zero until it first becomes positive again for the start of the next excursion. The *value *of an excursion is defined to be the peak score during the course of that particular excursion.

### Distribution of the Maximal Aggregate Score

For each value of *x*, the maximal aggregate score

Mn=max⁡1≤k≤nEk
 MathType@MTEF@5@5@+=feaafiart1ev1aaatCvAUfKttLearuWrP9MDH5MBPbIqV92AaeXatLxBI9gBaebbnrfifHhDYfgasaacH8akY=wiFfYdH8Gipec8Eeeu0xXdbba9frFj0=OqFfea0dXdd9vqai=hGuQ8kuc9pgc9s8qqaq=dirpe0xb9q8qiLsFr0=vr0=vr0dc8meaabaqaciaacaGaaeqabaqabeGadaaakeaacqWGnbqtdaWgaaWcbaGaemOBa4gabeaakiabg2da9maaxababaGagiyBa0MaeiyyaeMaeiiEaGhaleaacqaIXaqmcqGHKjYOcqWGRbWAcqGHKjYOcqWGUbGBaeqaaOGaemyrau0aaSbaaSqaaiabdUgaRbqabaaaaa@3E95@

satisfies

P(Mn>ln⁡nλ∗+x)≈1−exp⁡{−K∗e−λ*x},
 MathType@MTEF@5@5@+=feaafiart1ev1aaatCvAUfKttLearuWrP9MDH5MBPbIqV92AaeXatLxBI9gBaebbnrfifHhDYfgasaacH8akY=wiFfYdH8Gipec8Eeeu0xXdbba9frFj0=OqFfea0dXdd9vqai=hGuQ8kuc9pgc9s8qqaq=dirpe0xb9q8qiLsFr0=vr0=vr0dc8meaabaqaciaacaGaaeqabaqabeGadaaakeaacqWGqbaudaqadaqaaiabd2eannaaBaaaleaacqWGUbGBaeqaaOGaeyOpa4ZaaSaaaeaacyGGSbaBcqGGUbGBcqWGUbGBaeaaiiGacqWF7oaBdGaGKYbaaSqajaiPbGaGKIGaaiad0HSFxiIkaaaaaOGaey4kaSIaemiEaGhacaGLOaGaayzkaaGaeyisISRaeGymaeJaeyOeI0IagiyzauMaeiiEaGNaeiiCaaNaei4EaSNaeyOeI0Iaem4saS0aaWbaaSqabeaacWaDK63fIOcaaOGaemyzau2aaWbaaSqabeaacqGHsislcqWF7oaBdaahaaadbeqaaiadaYUGQaGkaaWccqWG4baEaaGccqGG9bqFcqGGSaalaaa@5979@

where λ* is the unique positive solution to the equation E(eλX1)
 MathType@MTEF@5@5@+=feaafiart1ev1aaatCvAUfKttLearuWrP9MDH5MBPbIqV92AaeXatLxBI9gBaebbnrfifHhDYfgasaacH8akY=wiFfYdH8Gipec8Eeeu0xXdbba9frFj0=OqFfea0dXdd9vqai=hGuQ8kuc9pgc9s8qqaq=dirpe0xb9q8qiLsFr0=vr0=vr0dc8meaabaqaciaacaGaaeqabaqabeGadaaakeaacqWGfbqrdaqadaqaaiabdwgaLnaaCaaaleqabaacciGae83UdWMaemiwaG1aaSbaaWqaaiabigdaXaqabaaaaaGccaGLOaGaayzkaaaaaa@34E3@ = *pe*^*λs *^+ *qe*^*λw *^= 1 and K* is a parameter given by an explicit series expansion (See [23]).

When *X *is an integer-valued variable of span *δ*, we have a simpler expression for K* ([23]):

exp⁡{−K+e−λ∗x}≤lim⁡inf⁡n→∞ P(Mn−ln⁡nλ*<x)≤lim⁡sup⁡n→∞ P(Mn−ln⁡nλ*<x)≤exp⁡{−K−e−λ*x},
 MathType@MTEF@5@5@+=feaafiart1ev1aaatCvAUfKttLearuWrP9MDH5MBPbIqV92AaeXatLxBI9gBaebbnrfifHhDYfgasaacH8akY=wiFfYdH8Gipec8Eeeu0xXdbba9frFj0=OqFfea0dXdd9vqai=hGuQ8kuc9pgc9s8qqaq=dirpe0xb9q8qiLsFr0=vr0=vr0dc8meaabaqaciaacaGaaeqabaqabeGadaaakqaaeeqaaiGbcwgaLjabcIha4jabcchaWjabcUha7jabgkHiTiabdUealnaaBaaaleaacqGHRaWkaeqaaOGaemyzau2aaWbaaSqabeaacqGHsisliiGacqWF7oaBcWaDez4fIOIaemiEaGhaaOGaeiyFa0NaeyizIm6aaCbeaeaacyGGSbaBcqGGPbqAcqGGTbqBcyGGPbqAcqGGUbGBcqGGMbGzaSqaaiabd6gaUjabgkziUkabg6HiLcqabaGccqWGqbaudaqadaqaaiabd2eannaaBaaaleaacqWGUbGBaeqaaOGaeyOeI0YaaSaaaeaacyGGSbaBcqGGUbGBcqWGUbGBaeaacqWF7oaBcWal0jOkaOcaaiabgYda8iabdIha4bGaayjkaiaawMcaaaqaaiabgsMiJoaaxababaGagiiBaWMaeiyAaKMaeiyBa0Magi4CamNaeiyDauNaeiiCaahaleaacqWGUbGBcqGHsgIRcqGHEisPaeqaaOGaemiuaa1aaeWaaeaacqWGnbqtdaWgaaWcbaGaemOBa4gabeaakiabgkHiTmaalaaabaGagiiBaWMaeiOBa4MaemOBa4gabaGae83UdWMamGdGcQcaQaaacqGH8aapcqWG4baEaiaawIcacaGLPaaaaeaacqGHKjYOcyGGLbqzcqGG4baEcqGGWbaCcqGG7bWEcqGHsislcqWGlbWsdaWgaaWcbaGaeyOeI0cabeaakiabdwgaLnaaCaaaleqabaGaeyOeI0Iae83UdWMamqhAcQcaQiabdIha4baakiabc2ha9jabcYcaSaaaaa@9178@

where

K−=λ*δeλ*δ−1K*, K+=λ*δ1−e−λ*δK*.
 MathType@MTEF@5@5@+=feaafiart1ev1aaatCvAUfKttLearuWrP9MDH5MBPbIqV92AaeXatLxBI9gBaebbnrfifHhDYfgasaacH8akY=wiFfYdH8Gipec8Eeeu0xXdbba9frFj0=OqFfea0dXdd9vqai=hGuQ8kuc9pgc9s8qqaq=dirpe0xb9q8qiLsFr0=vr0=vr0dc8meaabaqaciaacaGaaeqabaqabeGadaaakeaacqWGlbWsdaWgaaWcbaGaeyOeI0cabeaakiabg2da9maalaaabaacciGae83UdW2aaWbaaSqabeaacWaGulOkaOcaaOGae8hTdqgabaGaemyzau2aaWbaaSqabeaacqWF7oaBdaahaaadbeqaaiadacRGQaGkaaWccqWF0oazaaGccqGHsislcqaIXaqmaaGaem4saS0aaWbaaSqabeaacWaGilOkaOcaaOGaeiilaWIaeeiiaaIaem4saS0aaSbaaSqaaiabgUcaRaqabaGccqGH9aqpdaWcaaqaaiab=T7aSnacmskhaaWcbKaJKgacmsQamai1cQcaQaaakiab=r7aKbqaaiabigdaXiabgkHiTiabdwgaLnaaCaaaleqabaGaeyOeI0Iae83UdW2aiGjGCaaameqcycyaiGjGcWaGWkOkaOcaaSGae8hTdqgaaaaakiabdUealnaaCaaaleqabaGamaiYcQcaQaaakiabc6caUaaa@6263@

For the simple score scheme with values {-*m*, ..., -1, 0, 1} occurring with probabilities {*p*_-*m*_, ..., *p*_-1_, *p*_0_, *p*_1_} we have,

*K*_- _= (*e*^-λ* ^- *e*^-2λ*^) *E *(*Xe*^λ*X^).

We can set the left hand side of Equation (2) to some predetermined significance level, say *P *= 0.05 or 0.01, and solve for *x*. A segment with score exceeding MP=ln⁡nλ*+x
 MathType@MTEF@5@5@+=feaafiart1ev1aaatCvAUfKttLearuWrP9MDH5MBPbIqV92AaeXatLxBI9gBaebbnrfifHhDYfgasaacH8akY=wiFfYdH8Gipec8Eeeu0xXdbba9frFj0=OqFfea0dXdd9vqai=hGuQ8kuc9pgc9s8qqaq=dirpe0xb9q8qiLsFr0=vr0=vr0dc8meaabaqaciaacaGaaeqabaqabeGadaaakeaacqWGnbqtdaWgaaWcbaGaemiuaafabeaakiabg2da9maalaaabaGagiiBaWMaeiOBa4MaemOBa4gabaacciGae83UdW2aaWbaaSqabeaacWaAulOkaOcaaaaakiabgUcaRiabdIha4baa@3ADC@ is then said to be significant at the 100*P*% level.

In this paper, we use *K*_- _in place of K* in Equation (2) for a "conservative" estimate of the probability and *K*_+ _for a "generous" one.

We use Equation (2) with *P *= 0.05 and *P *= 0.01 to get *M*_0.05 _and *M*_0.01 _respectively. If the value of an excursion exceeds the critical value *M*_0.05 _(or *M*_0.01_), then the segment from the beginning of the excursion up to the base where the peak value is realized is said to be a high-scoring segment (HSS) significant at the 5% (or 1%) level.

### HSS Selection

For each of the genomic sequences listed in Table 1, we obtain a set of HSS, significant at the 5% (or 1%) level. In each set of HSS, it is common to find several of them located close to one another. We thus apply a filtering procedure so that, if this happens, we shall only select one of several neighboring excursions as a representative for that part of the genome. In fact, we first sort all the HSS according to their aggregate scores. Starting with the one with the highest value, say segment A, we 'discard' neighboring HSS that are within 2 map units of it. After that, we pick among the rest (not including segment A and the discarded HSS), the HSS with the next highest value, say segment B, and repeat the process. Only the representative segments A, B, and so forth, will be used in replication origin prediction.

## Results and Discussion

### HSS Tables and Excursion Plots

Table 2 lists the HSS for each herpesvirus in Table 1. We have also tried locating high-scoring segments by running the excursions from the 3' end to 5' end of the genome. The results obtained are not much different from the "vanilla" version (i.e., from 5' to 3').

**Table 2:** Herpesviruses : HSS at 5% level using the conservative bound.

**HSS**				**HSS**			
**Virus**	**Start**	**Peak**	**Value**	**Virus**	**Start**	**Peak**	**Value**
alhv1	*1204*	*1370*	*54*	ebv	*11854*	*11950*	*45*
	*32478*	*32850*	*48*		*77111*	*77150*	*24*
	*113630*	*113684*	*46*		*43158*	*43235*	*23*
	*85923*	*85992*	*45*	ehv1	*20348*	*20431*	*47*
	*72999*	*73115*	*44*		*134195*	*134276*	*36*
	125691	125726	31		*65055*	*65126*	*35*
athv3	8827	8892	40		*99301*	*99374*	*34*
bohv1	*100410*	*100484*	*26*		*11034*	*11141*	*32*
	*109702*	*109730*	*25*		*105796*	*105862*	*30*
	*128487*	*128515*	*25*		*73653*	*73746*	*27*
	*16593*	*16626*	*21*		*113818*	*113849*	*25*
	*113720*	*113738*	*18*		*149310*	*149341*	*25*
	*124479*	*124497*	*18*		110314	110352	23
	29	45	16		128924	128992	23
	58542	58569	15	ehv2	*160281*	*160518*	*102*
bohv4	60687	60826	35		*86522*	*86622*	*76*
bohv5	*68440*	*68507*	*49*		*53843*	*54012*	*61*
	*113549*	*113583*	*28*		*140661*	*140826*	*57*
	*129429*	*129463*	*28*		*4580*	*4655*	*51*
	*592*	*616*	*21*		*171454*	*171529*	*51*
	*86191*	*86215*	*21*		*95342*	*95440*	*50*
	*102074*	*102106*	*17*		*10772*	*10820*	*48*
	*92511*	*92535*	*15*		*39893*	*39977*	*48*
	*120935*	*120959*	*15*		*177646*	*177694*	*48*
	59921	59938	14		*113310*	*113399*	*47*
	17408	17433	13		*134709*	*134772*	*45*
	41883	41899	13		*166114*	*166207*	*42*
calhv3	*70131*	*70198*	*31*		*45831*	*45965*	*41*
ccmv	*50872*	*50973*	*50*		*15443*	*15482*	*39*
	*158344*	*158701*	*45*		*19722*	*19845*	*39*
	*95375*	*95603*	*39*		*182317*	*182356*	*39*
	*3519*	*3602*	*35*		*153977*	*154145*	*36*
	*24084*	*24156*	*33*		*123321*	*123362*	*35*
	*182982*	*183136*	*31*		*147222*	*147341*	*35*
	*14314*	*14370*	*23*		*34816*	*34884*	*29*
	*177170*	*177247*	*23*		*76380*	*76454*	*29*
	*189041*	*189075*	*22*		*103167*	*103223*	*29*
	147310	147384	20		*64344*	*64402*	*25*
cehv1	*116723*	*116836*	*53*		786	831	24
	*92092*	*92118*	*26*	ehv4	*109852*	*110086*	*60*
	*61680*	*61700*	*20*		*19878*	*19943*	*50*
	*132785*	*132805*	*20*		*132383*	*132462*	*49*
	*149415*	*149435*	*20*		*105284*	*105365*	*48*
	*52055*	*52075*	*17*		*23895*	*24016*	*43*
	*42984*	*43006*	*16*		*3984*	*4110*	*42*
	*11389*	*11407*	*15*		*73340*	*73509*	*37*
	24415	24441	14		*98849*	*98930*	*33*
cehv15	*11965*	*12011*	*28*		*46612*	*46674*	*32*
	114927	114988	19		10630	10697	31
cehv16	*92913*	*92940*	*23*		58833	58906	31
	*62970*	*62991*	*21*		82616	82701	31
	*133468*	*133489*	*21*		127230	127351	31
	*149813*	*149834*	*21*		112929	112967	29
	*8303*	*8331*	*20*		145082	145120	29
	*118685*	*118713*	*20*	gahv1	*24852*	*24890*	*30*
	*53056*	*53100*	*18*	gahv2	106724	106811	35
	*25423*	*25473*	*16*	gahv3	11168	11198	27
	*1717*	*1736*	*15*		122384	122414	27
	*114861*	*114890*	*15*		134414	134461	26
	*125280*	*125299*	*15*		162999	163046	26
	30975	30991	14		58953	58999	25
cehv2	*7681*	*7738*	*33*	hcmv	*3402*	*3542*	*41*
	*115791*	*115848*	*33*		*186855*	*186995*	*41*
	*61483*	*61503*	*20*		*16757*	*16915*	*35*
	*129527*	*129547*	*20*		*96685*	*96824*	*34*
	*144461*	*144481*	*20*		*11713*	*11808*	*32*
	*90857*	*90884*	*19*		*198116*	*198171*	*31*
	51884	51910	14		*173560*	*173599*	*30*
	93873	93887	14		*210724*	*210781*	*30*
	112292	112320	14		*26361*	*26475*	*27*
cehv7	86167	86296	37		108222	108303	24
cehv8	*149643*	*149720*	*33*		159296	159380	24
	*15671*	*15733*	*30*		71011	71055	23
	29233	29278	29		226192	226230	23
	163766	163806	28				
	177904	178092	28				
	89538	89589	27				
hcmv-m	*3798*	*3939*	*42*	mfrv	*128046*	*128640*	*114*
	*181238*	*181334*	*33*		*23139*	*23374*	*109*
	*97069*	*97206*	*32*		*2488*	*3068*	*106*
	*173950*	*173994*	*32*		*32573*	*33752*	*84*
	*216020*	*216077*	*30*		*64296*	*64454*	*62*
	*203400*	*203456*	*29*		*111496*	*111624*	*44*
	*17082*	*17297*	*26*		*72739*	*72809*	*43*
	*12060*	*12145*	*25*		*53766*	*53825*	*32*
	*157590*	*157726*	*25*		*69912*	*70061*	*32*
hhv6	*130410*	*130501*	*59*		*114828*	*114860*	*32*
	*3605*	*3712*	*51*	mmrv	*2388*	*2967*	*111*
	*154838*	*154945*	*51*		*23902*	*24187*	*108*
	*137079*	*137210*	*43*		*33761*	*35136*	*103*
hhv6b	*132997*	*133163*	*62*		*130346*	*131085*	*97*
	*139482*	*139569*	*51*		*65611*	*65853*	*56*
	3911	3988	37		*74140*	*74204*	*37*
	157232	157309	37		*71311*	*71462*	*31*
hhv7	*134169*	*134376*	*117*		*117507*	*117551*	*29*
	*128589*	*128984*	*70*		112930	113033	28
hhv8	*136287*	*136704*	*93*	muhv4	6000	6037	29
	*982*	*1125*	*44*	ohv2	*115365*	*115545*	*72*
	*58833*	*58906*	*28*		*126823*	*127116*	*68*
	23547	23598	27		*118943*	*118988*	*42*
	30712	30775	27		*72630*	*72699*	*36*
	119416	119467	27		*1269*	*1370*	*29*
	106412	106452	25		*27589*	*27633*	*29*
hsv1	*62465*	*62485*	*20*		76335	76370	26
	*35000*	*35034*	*19*		79158	79265	26
	*115242*	*115303*	*19*	oshv1	*73292*	*73460*	*64*
	*131990*	*132008*	*18*		*35416*	*35493*	*61*
	*144115*	*144142*	*18*		*146021*	*146164*	*55*
	*11705*	*11734*	*17*		*190174*	*190312*	*54*
	*52753*	*52818*	*17*		*195928*	*196026*	*54*
	96047	96069	16		*201648*	*201786*	*54*
	136146	136162	16		*23065*	*23135*	*50*
hsv2	*5584*	*5628*	*35*		*161395*	*161505*	*50*
	*121621*	*121665*	*35*		*2682*	*2735*	*49*
	*52978*	*53003*	*19*		*180276*	*180329*	*49*
	*91716*	*91747*	*19*		108068	108173	45
	*146600*	*146631*	*19*		171433	171549	44
	*95238*	*95256*	*18*		67872	67975	43
	*48761*	*48778*	*17*		114689	114763	42
	*62919*	*62939*	*17*	pshv1	*18751*	*18791*	*31*
	*132691*	*132711*	*17*		*121452*	*121486*	*31*
	*81195*	*81220*	*16*		*160685*	*160719*	*31*
	99337	99370	15		*130332*	*130365*	*27*
ichv1	*6068*	*6290*	*81*		*151806*	*151839*	*27*
	*121738*	*121960*	*81*		*23896*	*23942*	*22*
	*104134*	*104399*	*70*		134013	134049	21
	*17065*	*17333*	*58*		78233	78256	20
	*132735*	*133003*	*58*	rcmv	*150923*	*151612*	*92*
	*451*	*726*	*50*		*207600*	*207980*	*80*
	*116121*	*116396*	*50*		*143617*	*144150*	*74*
	*60752*	*60845*	*30*		*178241*	*178326*	*37*
	*42919*	*43007*	*28*		*214638*	*214702*	*37*
	20109	20187	24		*219069*	*219153*	*33*
	10016	10063	23		*201767*	*201885*	*28*
	125686	125733	23		*161797*	*161929*	*27*
mcmv	*155163*	*156341*	*125*		*171828*	*171870*	*27*
					24072	24108	21
	*161228*	*161391*	*40*	sahv2	28533	28613	45
	*115543*	*115640*	*37*	shv1	*63862*	*63892*	*24*
	*102865*	*102960*	*35*		*96251*	*96275*	*21*
	*79497*	*79573*	*34*		*114686*	*114715*	*20*
	*15628*	*15724*	*33*		*129607*	*129636*	*20*
	*144170*	*144290*	*33*		*50382*	*50407*	*19*
	*73525*	*73579*	*27*		*75955*	*75984*	*17*
	*39209*	*39248*	*24*		*16151*	*16172*	*15*
	*92997*	*93036*	*24*		*33045*	*33063*	*15*
	219239	219282	22		*109083*	*109098*	*15*
mehv1		NIL			*135503*	*135518*	*15*
					8432	8455	14
				thv	*168842*	*168927*	*25*
					*24153*	*24200*	*23*
					28257	28286	17
				vzv	*2574*	*2785*	*39*
					110195	110227	32
					119669	119701	32

Entries in *italics *are significant at 1% too.

**Figure 1:**
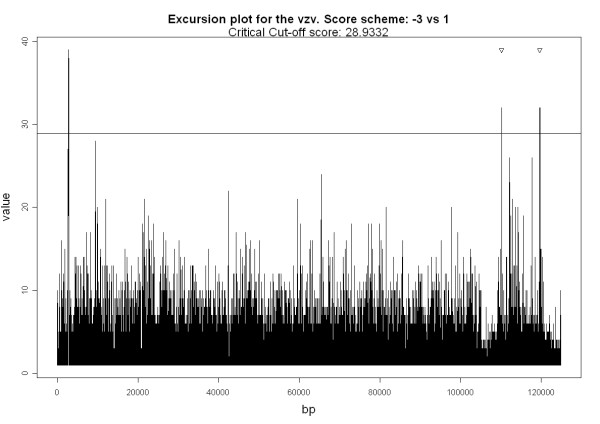
**The Excursion Plot of the vzv virus**. The horizontal line corresponds to the 5% significant level. The two triangles denote the locations of known replication origins of the vzv.

For visualizing the locations of the selected HSS relative to the entire genome, the excursion plot is a convenient tool. The excursion plot of the Human Herpesvirus 3 (vzv) is presented in Figure 1, where the AT excursion values are plotted against the bases along the genome. The general appearance of Figure 1 is typical of the excursion plots for all the herpesviruses analyzed. In the case of vzv, three peaks with excursion values exceeding the 5% significance level are observed. Two of these peaks are close to the centers of the only two known replication origins of vzv (see Table 3).

**Table 3:** Prediction results at 5% level using the conservative bound.

**Nearest HSS**					
**Virus**	**Ori Center**	**Start**	**Peak**	**Value**	**Prediction**
bohv1	111190	109702	109730	25	Yes
bohv1	127028	128487	128515	25	Yes
bohv4	97996.5	60687	60826	35	No
bohv5	113312	113549	113583	28	Yes
bohv5	129701	129429	129463	28	Yes
cehv1	61690.5	61680	61700	20	Yes
cehv1	61893.5	61680	61700	20	Yes
cehv1	132795.5	132785	132805	20	Yes
cehv1	132998.5	132785	132805	20	Yes
cehv1	149425.5	149415	149435	20	Yes
cehv1	149628.5	149415	149435	20	Yes
cehv16	62981	62970	62991	21	Yes
cehv16	133479	133468	133489	21	Yes
cehv16	149824	149813	149834	21	Yes
cehv2	61493.5	61483	61503	20	Yes
cehv2	129537.5	129527	129547	20	Yes
cehv2	144471.5	144461	144481	20	Yes
cehv7	109636.5	86167	86296	37	No
cehv7	118622.5	86167	86296	37	No
ebv	8313.5	11854	11950	45	No
ebv	40797	43158	43235	23	Yes
ebv	143825.5	77111	77150	24	No
ehv1	126262.5	128924	128992	23	Yes
ehv4	73909.5	73340	73509	37	Yes
ehv4	119471.5	112929	112967	29	No
ehv4	138577.5	132383	132462	49	No
gahv1	24871.5	24852	24890	30	Yes
hcmv	93923.5	96685	96824	34	Yes
hhv6	67805	130410	130501	59	No
hhv6b	69160.5	132997	133163	62	No
hhv7	66991.5	128589	128984	70	No
hsv1	62475	62465	62485	20	Yes
hsv1	131999	131990	132008	18	Yes
hsv1	146235	144115	144142	18	Yes
hsv2	62930	62919	62939	17	Yes
hsv2	132760	132691	132711	17	Yes
hsv2	148981	146600	146631	19	Yes
rcmv	77318	24072	24108	21	No
shv1	63878	63862	63892	24	Yes
shv1	114701	114686	114715	20	Yes
shv1	129901	129607	129636	20	Yes
vzv	110218.5	110195	110227	32	Yes
vzv	119678.5	119669	119701	32	Yes

For each replication origin, we list the high-scoring segment (at 5% level) closest to it. When the peak of a high-scoring segment is less than 2 map units away from the center of a replication origin, we say that our method has correctly predicted that particular replication origin.

### Prediction Performance

The high-scoring segments are checked against known replication origins in herpesviruses to evaluate their performance as a prediction tool. Table 3 lists all the known replication origins for the herpesviruses in Table 1. These origins are reported either in published literature or GenBank annotations. For each replication origin, we list the HSS (at 5% level) closest to it. For this table we had used the "conservative" estimate for the value of K* (See Equations (2) and (3)). When the peak of an HSS is less than 2 map units (one map unit is one percent of the genome length) away from the center of a replication origin, we say that our method has correctly predicted that particular replication origin. From Table 3, we see that of the 43 replication origins known, compiled from literature or annotations, 32 of them are close to HSS that have been identified.

We had also tried using the "generous" estimate for K* at the 5% and 1% level of significance. Table 4 gives a summary of the performance of our prediction scheme when those bounds were used. The first two columns of the table gives the *sensitivity *level and *positive prediction value *of our scheme. Sensitivity refers to the percentage of replication origins predicted by our method, and PPV (positive predictive value) the proportion of HSS that correctly predict replication origins. *APD (average predictive distance)*, given in map units (± one standard deviation), shows the average of the distances (in map units) between the center of each replication origin and the HSS that predicts it. Note that the APD values say that on average, when a prediction by an HSS is successful, the replication origin is about 0.35 map units away from it. We have also done some simple analysis of the location of the center of each replication origin with respect to the HSS closest to it. We count the number of times the center of replication origin falls within the left, right or center of the HSS. The columns %L, %R, and %C in Table 4 give these proportions. Our results show that the origin falls within the center of the HSS half the time.

**Table 4:** Prediction Performance Summary.

**Significance**	**Sensitivity**	**PPV**	**APD**	**%L**	**%R**	**%C**
**5% (C)**	74%	22%	0.34 ± 0.57	16%	31%	53%
**5% (G)**	86%	17%	0.35 ± 0.53	24%	30%	46%
**1% (C)**	67%	25%	0.31 ± 0.52	14%	34%	52%
**1% (G)**	74%	18%	0.34 ± 0.57	16%	31%	53%

(C) indicates that the "Conservative" bound is used while (G) indicates that the "Generous" bound is used. Sensitivity refers to the percentage of replication origins predicted by our method, and PPV (positive predictive value) the proportion of HSS that correctly predict replication origins. *APD (average predictive distance)*, given in map units (± one standard deviation), shows the average of the distances between the center of each replication origin and a HSS that predicts it in map units. %*L*, %*R *and %*C *count the number of times the center of replication origin falls within the left, right or center of the HSS.

### Comparison with Other Approaches

**Figure 2:**
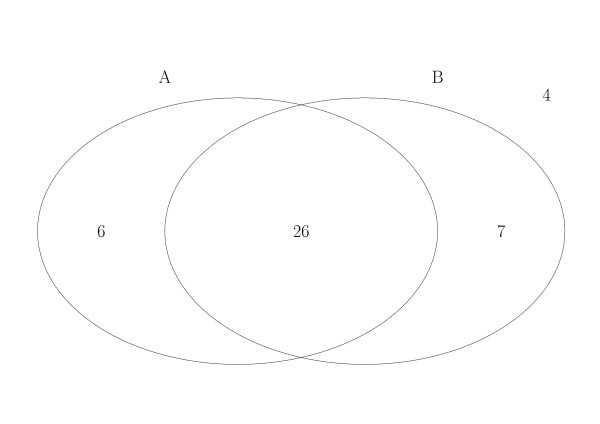
**Predictions of AT excursion and BWS_1_**. In this figure, the set *A *consists of origin replications predicted by the AT excursion method and *B *consists of those predicted by the BWS_1 _method. A ⋂ B^*C *^= {cehv7_1_, cehv7_2_, ehv4_1_, hsv2_1_, hsv2_2_, hsv2_3_}, A^*C *^⋂ B = {cehv16_2_, cehv16_3_, ebv_1_, ebv_3_, hhv6, hhv6b, rcmv}, (A ⋃ B)^*C *^= {bohv4, ehv4_2_, ehv4_3_, hhv7}. The rest of the replication origins (26 of them) are predicted by both methods. Note that for viruses with several known replication origins, such as the hsv2, which has three (see Table 3), we denote the replication origins as hsv2_1_, hsv2_2_, hsv2_3_, etc.

How does the AT excursion method compare with the sliding window approach using palindrome based scoring schemes previously presented in [12]? Since the BWS_1 _scheme has been shown to perform best among the various palindrome based schemes, we have examined the numbers of replication origins correctly predicted by AT excursion and by BWS_1_. The results are summarized in Figure 2.

The majority of the 43 known origins in the herpesviruses listed in Table 1 are predicted by both methods and most of the remaining ones are predicted by one method or the other. Only four of the origins fail to be predicted by either method. This suggests that the AT excursion method and the BWS_1 _scheme complement each other very well.

There are certain advantages in the AT excursion approach over BWS_1_. First, AT excursion does not require any sequence specific parameters to be prescribed by the user. It is window size free because it does not require any sliding window to measure AT concentration. Moreover, while the palindrome based methods require the specification of a minimal palindrome length before the analysis can be carried out, no such parameter is needed for AT excursion. Second, the AT excursion method is statistically based, as the probabilistic distribution has already been established [20-22]. This allows the statistical significance for HSS be evaluated easily.

**Figure 3:**
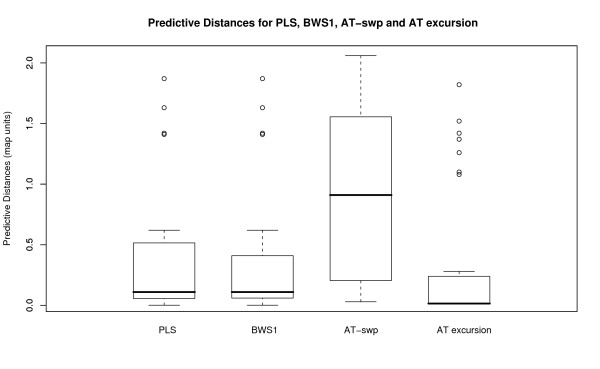
**Predictive Distances for PLS, BWS_1_, AT-swp and AT excursion**. These boxplots show the predictive distances for PLS, BWS_1_, AT-swp and AT excursion.

We also note that the more elaborate AT excursion approach performs better than the simpler procedure of measuring the percentage of A and T bases on a sliding window in terms of number of correct predictions and the proximity of these predictions to the true origins. Out of the 43 known replication origins for the herpesviruses in Table 1, 32 are correctly predicted by AT excursion but only 28 by AT sliding window plot. Furthermore, the boxplots of the predictive distances (Figure 3) of the AT excursion approach suggests that the predictions given by the AT excursion approach are much closer to known replication origins as compared to those of the AT sliding window plot approach. (In fact, the predictive distances of the AT excursion approach compared to that of the PLS and BWS_1 _approaches mentioned in [12] are observably shorter. See Figure 3.) This suggests that the excursion values might more correctly capture the essence of A/T abundance variation along genomic sequences.

### Herpesvirus Replication Origins Alignment and Motif Finding

One might ask whether or not the nucleotide sequences around replication origins in various viruses of the same family share sufficient similarities so that the origins can be identified by sequence alignments and motif finding techniques. We therefore extracted the nucleotide sequences of the known herpesvirus origins according to their documented locations for closer examination. These sequences are available as supplementary materials on the companion website. A multiple alignment using CLUSTAL W [24] and motif searches using MEME and MAST [25,26] have been conducted for the herpesvirus origin sequences. No significant sequence similarity or common motif pattern across all the origin sequences has been found, agreeing with the findings of [11,13,14].

What if we first classify these nucleotide sequences according to some classification schemes, will the members within each class share noticeable sequence similarities? We classified the origins according to (i) the sub-family of the virus (herpesviruses are classified into the alpha, beta, and gamma sub-families by their biological properties [27]), (ii) the type of origin (i.e., whether the origin is a oriL, oriLyt or oriS). We ran MEME and MAST separately on the sequences in each sub-family/type of origins to detect common motif patterns. From the outputs under classification (i), we note that the origins from the alpha sub-family can be further divided into two groups. Each group has a common motif pattern across its members. For the beta and gamma sub-families, no distinct patterns can be found. However, the rcmv and ebv origins contain many repeat patterns. For classification (ii), we find that both the oriL and oriLyt origins contain sequence motifs common to a number of their members. No motif was found for oriS sequences. The results of our motif search are made available in the supplementary materials.

Although our investigations are preliminary, the motifs found in these subsets of herpesvirus genomes may suggest new information that can be incorporated into the replication origin prediction procedures.

### Other Families of Viruses

Aside from the herpesviruses, we have also applied the AT excursion method to search for HSS in the poxviruses and iridoviruses. These two viral families are chosen because, like the herpesviruses, they are large, complex dsDNA viruses with no RNA stage. Their genome lengths are also similar in magnitude to those of the herpesviruses.

Poxviruses infect a large variety of animal species that gather in swarms and herds (e.g., mosquitoes, cows). Smallpox is a major disease caused by the variola virus, a member of the poxvirus family. Smallpox was eradicated in 1977 by preventive inoculations with cowpox or vaccinia viruses through the dedicated efforts of the World Health Organization and many individuals. In the recent few years, as the threat of the variola virus being used as a biological weapon is raised, there is growing interest in further studying poxviruses for biodefense purposes [28,29]. Iridoviruses are found in a variety of fish, amphibians, and reptiles. Some iridoviruses have been associated with serious diseases (e.g., viral erythrocytic necrosis of salmonids), while others have only been found in apparently healthy animals (e.g., goldfish iridovirus). Iridovirus infection is considered a serious concern in modern aquaculture, fish farming, and wildlife conservation [30].

Amongst these two families, only one genome, namely the Chilo iridescent virus, has documented replication origin locations [31]. Our method has correctly predicted one of these locations. Due to the lack of confirmed origin locations, prediction accuracy cannot be tested on these families. Nevertheless, our predictions may assist researchers to investigate these viruses experimentally to identify and confirm the exact locations of replication origins in their genomes. We have, therefore, made our prediction results available at [1].

### AT excursion applied to larger genomes

To gauge whether the AT excursion approach can potentially be generalized to predict replication origins for non-viral genomes, we apply it to several archaeal and bacterial genomes which have been previously analyzed. From [4,5,32] we are able to compile a list of 15 known or suggested replication origins (11 known, 4 suggested). Using the AT excursion method, we manage to correctly predict 9 of the replication origins (6 known, 3 suggested). Although our studies are preliminary, the results show that the AT excursion method can work reasonably well even on larger genomes.

## Conclusion

This paper introduces the AT excursion method to quantify local AT abundance in genomic sequences. The simple and intuitive idea of locating regions with high AT content as potential replication origin sites proves to be effective in identifying several replication origins not previously predicted. This shows that the AT excursion approach is a valuable addition to existing prediction tools. However, we have also observed that quite a number of the statistically significant HSS found by AT excursions are not close to replication origins. Whether these HSS correspond to other important functional sites in the genomic sequences remains an interesting question to be investigated.

The availability of statistical significance criteria and the independence of ad hoc parameters like the minimal palindrome length and sliding window size make the AT excursion method particularly easy to apply to those viral genomes where no replication origin information in similar and related genomes is available. On the other hand, if such information is available, the AT excursion method is not capable of taking advantage of it. To address this issue, machine learning approaches (e.g., neural networks and support vector machines), which better allow us to use knowledge in related genomes, are currently being explored. We anticipate that a combination of score based statistics with machine learning approaches will provide a highly accurate prediction tool set for replication origins.

## Authors' contributions

DC participated in the design of the study and performed the data and statistical analysis. KPC and MYL conceived the study, and participated in its design and coordination. All authors contributed to writing, reading and approving the final manuscript.
